# Macromolecular Crystallography and Structural Biology Databases at NIST

**DOI:** 10.6028/jres.106.062

**Published:** 2001-12-01

**Authors:** Gary L. Gilliland

**Affiliations:** Center for Advanced Research in Biotechnology of the University of Maryland Biotechnology Institute and the National Institute of Standards and Technology, 9600 Gudelsky Drive, Rockville, MD 20850

**Keywords:** macromolecular crystallography, neutron crystallography, protein crystallography, proteins, structural biology databases, x-ray crystallography

## Abstract

In the late 1970s, macromolecular crystallography at NIST began with collaboration between NIST and NIH to establish a single-crystal neutron diffractometer. This instrument was constructed and employed to solve a number of crystal structures: bovine ribonuclease A, bovine-ribonuclease-uridine vanadate complex, and porcine insulin. In the mid 1980s a Biomolecular Structure Group was created establishing NIST capabilities in biomolecular singe-crystal x-ray diffraction. The group worked on a variety of structural problems until joining the NIST/UMBI Center for Advanced Research in Biotechnology (CARB) in 1987. Crystallographic studies at CARB were then focused on protein engineering efforts that included among others chymosin, subtilisin BPN*'*, interleukin 1β, and glutathione S-transferase. Recently, the structural biology efforts have centered on enzymes in the chorismate metabolic pathways involved in amino acid biosynthesis and in structural genomics that involves determining the structures of “hypothetical” proteins to aid in assigning function. In addition to crystallographic studies, structural biology database activities began with the formal establishment of the Biological Macro-molecule Crystallization Database in 1989. Later, in 1997, NIST in partnership with Rutgers and UCSD formed the Research Collaboratory for Structural Bioinformatics that successfully acquired the Protein Data Bank. The NIST efforts in these activities have focused on data uniformity, establishing and maintaining the physical archive, and working with the NMR community.

## 1. Introduction

Structural biology studies began at NIST in the late 1970s when it was recognized that neutron diffraction methods could be used to obtain novel information about the atomic structure of macromolecules, especially in its ability to elucidate hydrogen atom positions. NIH and NBS established a collaborative arrangement to develop macromolecular neutron crystallographic capabilities. Early work by Dr. John Norvell and later by Dr. Alexander Wlodawer resulted in the development and implementation of a neutron diffractometer with a linear detector specifically designed for collecting diffraction data from crystals of biological macromolecules [1]. The availability of the neutron diffractometer led to the determination of a number of protein structures. The requirements for these studies included protein crystals with relatively small unit cells, because of the diffraction data resolution requirements of the linear detector, and extremely large crystals (several millimeters in each dimension), because of the weak flux of the neutron beam [2].

In the mid 1980s the Biomolecular Structure Group was created in the Chemical Thermodynamics Division of the Center for Chemical Physics at NBS. Dr. Alexander Wlodawer who had been involved in establishing the NBS neutron diffractometer led this effort. This group established the first single-crystal macromolecular x-ray crystallographic laboratory at NBS. A number of important crystallographic studies were undertaken, many of which were completed prior to the incorporation of the group into the Center for Advanced Research in Biotechnology (CARB). This Center was established in the late 1980s when NIST began a long-term partnership with the University of Maryland. CARB was subsequently included as one of the Centers of the University of Maryland Biotechnology Institute (UMBI). The NIST crystallographic studies were focused on protein engineering. A number of productive structural investigations of proteins of industrial importance were undertaken, e.g., subtilisin [3], chymosin [4], and interleukin-1β [5]. As the CARB structural biology program matured, numerous other projects developed and were completed that have made significant contributions to understanding of how protein structure relates to function. Investigations of glutathione S-transferase [6], hemoglobin [7], uracil N-glycosylase [8], chorismate metabolism enzymes [9], and hypothetical protein targets associated with a structural genomics program [10] are representative of these efforts.

In addition to macromolecular crystallography, NIST staff members have been involved in the development and implementation of two important structural biology databases, the NIST/CARB Biological Macromolecule Crystallization Database [11] and the Protein Data Bank [12]. These efforts have involved collaborations with other laboratories and have been and continue to be important resources for the structural biology and other research communities.

The NBS/NIST structural biology efforts have been extremely productive over the years and have involved many NBS/NIST and CARB scientists, their collaborators, and numerous guest workers. Below the NBS/NIST history and achievements in structural biology are highlighted.

## 2. Neutron Diffraction Studies

### 2.2 Protein Structure Determinations

The neutron structures of bovine pancreatic ribonuclease A [13], a uridine vanadate-ribonuclease A complex [14], porcine 2 Zn insulin [15], and bovine pancreatic trypsin inhibitor [16] were all determined using data collected with the NBS neutron diffractometer. All four of these structures were refined using a joint x-ray and neutron procedure developed by Alex Wlodawer and Wayne Hendrickson [17]. Each of the structural investigations added important new information about how the structure relates to function and/or about our understanding of the general principles of protein structure.

The initial neutron studies of ribonuclease A produced difference Fourier maps at 2.8 Å resolution with phases that were derived from a model resulting from the joint refinement of neutron and x-ray data at 2.8 Å and 2.0 Å resolution, respectively [18]. These difference maps established the orientations of His12, His48, and His119 side chains for the first time. The orientation of His48 assumed during the refinement of the x-ray model at 2.5 Å resolution was confirmed, whereas the two active site histidines had to be rotated around CB-CG bonds in order to agree with the difference maps. In the final model, His12 is hydrogen bonded to the carbonyl oxygen of Thr45 and to the oxygen of the inorganic phosphate, and His119 forms a hydrogen bond with another oxygen of the phosphate and to the oxygen OD1 of Asp121.

The structure of ribonuclease A was refined jointly with the neutron and x-ray data extending to 2.0 Å [13]. The results of an earlier x-ray refinement provided the starting model [19]. The joint refinement resulted in the reorientation of a number of side chains, including the catalytically active Lys41, which is now thought to form a salt link to the phosphate. Major modifications to the early bound-solvent model were necessary. The refinement of atomic occupancies with only the neutron data provided the first information about amide hydrogen-deuterium exchange. Surprisingly, 28 of the 120 peptide amide hydrogen atoms were found to be fully or partially protected from exchange after a year of soaking the crystal in a D_2_O-containing mother liquor [20]. Most of the protected hydrogen atoms were involved in hydrogen bonds with main-chain carbonyl groups especially those that were part of the secondary structure. For example, residues 11–13 of the N-terminal α-helix were protected, as well as those in a contiguous region of the β-sheet containing residues 75, 106–109, 116, and 118, indicating their low flexibility and the lack of accessibility to solvent.

A complex of RNase A with a transition-state analog, uridine vanadate, was also studied using a combination of neutron and x-ray diffraction techniques [14]. The results provided the first structural information on RNase A with a bound transition-state analog. The vanadium atom occupies the center of a distorted trigonal bipyramid, with the ribose oxygen O2*'* at the apical position. Contrary to expectations based on the straightforward interpretation of the known in-line mechanism of action of RNase, nitrogen NE2 of His12 was found to form a hydrogen bond to the equatorial oxygen O8, while nitrogen NZ of Lys41 makes a clear hydrogen bond to the apical oxygen O2*'*. Nitrogen ND1 of His119 appears to be within a hydrogen-bond distance of the other apical oxygen, O7. Two other hydrogen bonds between the vanadate and the protein are made by nitrogen NE2 of Glu11 and by the amide nitrogen of Phe120. The observed geometry of the complex may necessitate reinterpretation of the mechanism of action of RNase.

A structural investigation of porcine 2 Zn insulin was also completed using the joint neutron/x-ray restrained-least-squares refinement procedure [15]. Neutron diffraction data to 2.2 Å resolution and x-ray data to 1.5 Å resolution were used in the study. As in the earlier studies, neutron diffraction data was obtained from a single crystal soaked in a mother liquor containing D_2_O. Surprisingly, when the protonation state of the individual amino acid residues was examined, no D atoms were found between the GluB13 carboxylates that make an intermolecular contact, suggesting a nonbonded interaction rather than the predicted hydrogen bond. Regions in the center of the B helices had unexchanged peptide-bond amide groups.

The structure of form II crystals of bovine pancreatic trypsin inhibitor was also determined using a joint refinement using both the neutron and x-ray data [16]. Crystallographic *R* factors for the final model were 0.197 for the 1.8 Å neutron data and 0.200 for the x-ray data extending to 1 Å resolution. The resulting structure was very similar to that of crystal form I (r.m.s. deviation for main chain atoms was 0.40 Å); nevertheless larger deviations were observed in particular regions of the chain. Twenty out of 63 ordered water molecules occupy similar positions (deviation less than 1 Å) in both models. Eleven amide hydrogen atoms were protected from exchange after three months of soaking the crystals in deuterated mother liquor at pH 8.2. Their locations were in excellent agreement with the results obtained by two-dimensional nuclear magnetic resonance, but the rates of exchange are much lower in the crystalline state.

## 3. Biomolecular Structure Group

The Biomolecular Structure Group of the Chemical Thermodynamics Division of the Center for Chemical Physics at NBS carried out a number of seminal crystallographic investigations of biological macromolecules. Dr. Alexander Wlodawer, the group leader and other group members carried out further studies on ribonuclease A [21–25], 2 Zn insulin [15], and pancreatic trypsin inhibitor [26–27]. New studies of bovine chymosin [4], interleukin-1β [28], and a DNA 15-mer duplex with mispaired bases [29] were initiated. These studies were completed when the group members moved to CARB or elsewhere. Dr. Irene Weber continued her investigation of the catabolite gene activator protein that she had started during her postdoctoral studies with Dr. Thomas Steitz at Yale [30–32]. Highlights of a number of these studies are presented below.

**Fig. 1:**
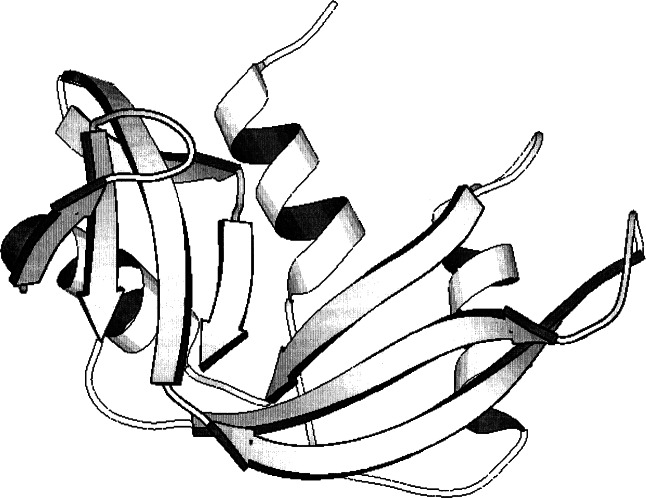
The 1.26 Å structure of bovine phosphate-free ribonuclease A (PDB entry 7rsa) [22]. The backbone fold is shown with the α-helix and β-strand secondary structure elements are shown as tightly coiled ribbons and arrows, respectively.

One focus of the Biomolecular Structure Group was the continued structural investigation of bovine ribonuclease A. The efforts involved collaboration with investigators at Genex Corporation and the University of Goteborg, Sweden. A fragment of a large crystal grown for neutron diffraction studies and the availability of one of the first commercially produced x-ray area-detector system, a Xentronics^1^ multiwire image proportional counter, led to one of the highest-resolution datasets for an enzyme, 1.26 Å resolution [21–23], at this time. The refined structure of phosphate-free bovine ribonuclease A consisted of all atoms in the polypeptide chain including hydrogens, 188 water sites with full or partial occupancy, and a single molecule of 2-methyl-2-propanol (Fig. 1). Thirteen side chains were modeled with two alternate conformations. These residues are widely distributed over the protein surface, but only one of them, Lys61, is involved in crystal packing interactions. For three of the residues, Val43, Asp83, and Arg85, two correlated conformations are found. Major changes to the active site include the addition of two waters in the phosphate-binding pocket, disordering of Gln11, and tilting of the imidazole ring of His119. This high-resolution structural study provided many new important details of how the structure of this enzyme relates to its function.

Another ongoing structure determination at this time was of a new crystal form, form III, of bovine pancreatic trypsin inhibitor [26]. The structure was solved by molecular replacement using the coordinates of forms I and II and the x-ray data extending to 1.7 Å resolution. The final model includes 73 water molecules and one phosphate group bound to the protein. Surprisingly, sixteen water molecules were found to occupy approximately the same positions in all three crystal forms, indicating an important role in the structure of the protein molecule. This structure led to one of the first detailed structural comparison of two high-resolution structures of bovine pancreatic trypsin inhibitor determined from two distinct crystal forms [27]. One of the structures was a result of a new least-squares x-ray refinement of data from crystal form I, while the other was the joint x-ray/neutron structure of crystal form II. The molecules showed an overall root-mean-squares deviation of 0.40 Å for the atoms in the main chain, while the deviations for the side-chain atoms are 1.53 Å. The latter number decreases to 0.61 Å when those side-chains that adopted drastically different conformations are excluded from comparison. The discrepancy between atomic temperature factors in the two models was 6.7 Å^2^, while their general trends are highly correlated. About half of the solvent molecules occupy similar positions in the two models, while the others are different. As expected, solvent molecules with the lowest temperature factors were the most likely to be common in the two crystal forms.

As mentioned above, Dr. Irene Weber continued her structural investigation of the *Escherichia coli* catabolite gene activator protein (CAP). CAP in the presence of cAMP stimulates transcription from several operons in *Escherichia coli*. In addition to extending the refined structure to 2.5 Å resolution [30], she initiated structural studies of variants that had novel properties. Crystal structure of a cyclic AMP-independent mutant of catabolite gene activator protein in which Ala144 is replaced by threonine was determined at 2.4 Å resolution [31]. This mutant lacks adenylate cyclase activity, but it does have a CAP phenotype; in the absence of cAMP it is able to express genes that normally require cAMP. The structural analysis revealed the two alanine to threonine sequence changes in the dimer, and also a change in the orientation of Cys178 in one of the subunits. Small changes in the conformation included concerted motions in the small domains in the hinge between the two domains and in an adjacent loop between beta-strands 4 and 5. The mutation at residue 144 apparently causes changes in the position of some protein atoms that are distal to the mutation site.

This Thr144Ala CAP variant is activated by analogues of cAMP, such as adenosine, which do not activate the wild-type protein. Crystals of the variant grown as a complex with cAMP were soaked in a solution of 10 mM adenosine, and x-ray diffraction data were measured to 3.5 Å resolution [32]. Adenosine was preferentially substituted for cAMP in only one of the two CAP subunits (in the “closed” conformation). Surprisingly, adenosine is not bound in exactly the same position as cAMP; the 5*'*-OH of adenosine is in a new position that allows formation of two hydrogen bonds with Ser-83, replacing two of the three interactions of the phosphate of cAMP with Arg-82 and Ser-83. This may help to explain the protein’s novel behavior.

## 4. Center for Advanced Research in Biotechnology (CARB)

NIST staff were formally assigned to CARB in 1987, and they moved to the current CARB research laboratories at the Shady Grove Campus of the University of Maryland in late 1989. The macromolecular crystallography efforts at CARB furthered the efforts started earlier at NIST in determining the structures of recombinant human interleukin-1β [5,33] and recombinant bovine chymosin [4,34–36]. In addition, new programs in protein engineering [37] of subtilisin BPN’ [3,38–50] and hemoglobin [7,51–56] were carried out as well as detailed structural investigations of a number of enzymes that included ribonuclease [57–64], several glutathione S-transferases [6,65–82], uracil DNA glycosylase [8,83–84], threonine deaminase [85–86], and nucleoside diphosphosphate transferase [87–88]. Several other structural investigations were also undertaken and completed [89–92]. This work was carried out by a group of NIST and University of Maryland Biotechnology Institute (UMBI) scientists and guest researchers led by Gary Gilliland. The NIST staff included Travis Gallagher, Neil Clarke, Jane Ladner, and Gregory Vasquez. Guest workers included L. Anders Svennsson (University of Goteborg, Sweden), Igor Pechik (Englehardt Institute of Molecular Biology, Moscow, Russia), Natalia Andreeva (Englehardt Institute of Molecular Biology, Moscow, Russia), Orna Almog (Israel), Richard Armstrong (University of Maryland/Vanderbilt) and Adela Rodriquez (Institute de Quimica, Mexico). The UBMI staff included B. Veerapandian, Xinhua Ji, Gaoyi Xiao, Maria Tordova, Reetta Raag and Jonathan Dill.

### 4.1 Interleukin-1β

One of the first structural biology efforts at CARB involved the crystal structure determination of recombinant human interleukin-1β (IL-1β) [28]. Interleukin-1β belongs to the cytokine family of cellular mediators. The cytokine structure was determined at 2.0 Å resolution and refined to a crystallographic *R*-factor of 0.19 [5]. The framework of this molecule consists of 12 anti-parallel β-strands exhibiting pseudo-3-fold symmetry. Six of the strands make up a β-barrel with polar residues concentrated at either end. Analysis of the three-dimensional structure, together with results from site-directed mutagenesis and biochemical and immunological studies, suggest that the core of the β-barrel plays an important functional role. A large patch of charged residues on one end of the barrel was proposed as the binding surface with which IL-1β interacts with its receptor.

The crystallographic data from this study was used in a joint refinement of a macromolecule against both x-ray crystallographic and NMR observations [33]. This collaborative work with NIH resulted in the first successful refinement of this type. The model of interleukin-1β derived by the joint x-ray and NMR refinement was shown to be consistent with the experimental observations of both methods and to have a crystallographic *R* value and geometrical parameters that are of the same quality as or better than those of models obtained by conventional crystallographic studies. The few NMR observations that are violated by the model serve as an indicator for genuine differences between the crystal and solution structures. The joint x-ray-NMR refinement can resolve structural ambiguities encountered in studies of multi-domain proteins, in which low- to medium-resolution diffraction data can be complemented by higher resolution NMR data obtained for the individual domains.

### 4.2 Chymosin

**Fig. 2:**
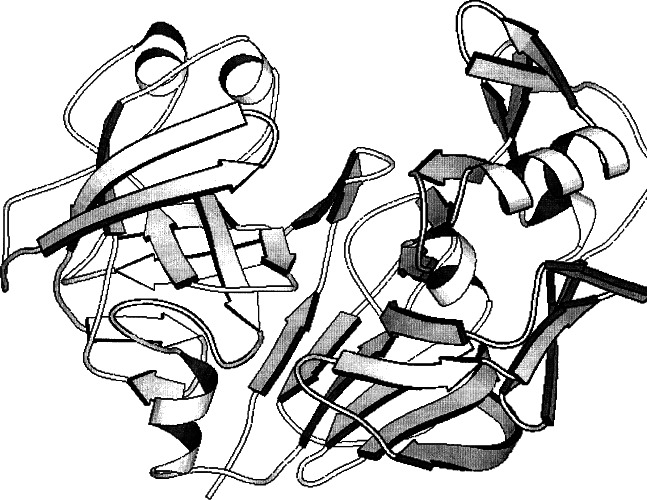
The 2.3 Å structure of bovine chymosin (PDB entry 1cms) [4]. The backbone fold is shown with the α-helix and β-strand secondary structure elements are shown as tightly coiled ribbons and arrows, respectively.

The crystal structure of recombinant bovine chymosin, which was cloned and expressed in *Escherichia coli*, was determined at 2.3 Å resolution (see Fig. 2) [4]. The enzyme has an irregular shape with approximate dimensions of 40 Å × 50 Å × 65 Å. The secondary structure consists of parallel and antiparallel β-strands with a few short α-helices. The enzyme has N- and C-terminal domains that are separated by a deep cleft containing the active aspartate residues Asp34 and Asp216. The amino acid residues and waters at the active site form an extensive hydrogen-bonded network that maintains the pseudo 2-fold symmetry of the entire structure. A comparison of recombinant chymosin with other acid proteases reveals the high degree of structural similarity with other members of this family of proteins as well as the subtle differences that make chymosin unique. The chymosin structure has Tyr77 occluding the S1/S3 substrate binding pockets suggesting that the enzyme is self-inhibited [34]. An analysis of this structure in conjunction with its comparison with pepsin has shown that this is most probably an intrinsic property of the enzyme. It also indicates that chymosin’s substrate specificity may be dependent upon the ability of the substrate to displace the tyrosine ring from the binding pockets. This analysis also implies that active and self-inhibited forms of other aspartic proteinases can exist in solution helping to explain the results of kinetic studies of these enzymes.

Attempts at obtaining crystals with bound substrate analogs that are suitable for diffraction studies were unsuccessful. Therefore, substrate binding was examined by model building substrates and substrate analogs into the active site cleft of the structure [35]. The model complexes were compared with the structures of inhibitor-aspartic proteinase complexes that have been previously reported. The results indicated that there are valid reasons why the natural substrate, kappa-casein, binds and is cleaved between positions 105–106. The positively charged histidine residues (98, 100, and 102) of κ-casein, which are located prior to the cleavage site, appear to be able to interact with negatively charged residues of chymosin, which are quite distant from the active site. These residues include Glu288, Asp279, and Glu280 of chymosin. The latter two residues are approximately 20 Å and 25 Å from the center of the active site. These studies also suggested that the difference in activities of the A and B isozymes of chymosin may be due to the increased binding affinity of the substrate as a result of strong electrostatic interactions with Asp244 of chymosin and positively charged His102 of the substrate. It was observed from the structure that the N-terminal domain has a smaller net negative charge than the C-terminal domain. This is due to a patch of positive charges on the surface located in the region from residues 48 to 62. Electrostatic calculations in which overall dipole moments were estimated for each of the eukaryotic aspartic proteinases have been performed.

The data used in the structure determination of chymosin was used to test an *ab inito* crystallographic phasing method [36]. An efficient algorithm for the determination of an all positive electron-density distribution that agrees with observed structure amplitudes was used to determine the phases of x-ray diffraction data from chymosin. A systematic procedure for testing the signs of centric reflections, using the total entropy of the map as a figure of merit, was used to produce a low-resolution map. The phases of acentric and additional centric reflections were then chosen by adding them to the map with various possible phases and computing the total entropy of the resulting map. Of 159 centric reflections whose phases were chosen by this procedure, 141 had the same phase as in the refined structure. The median absolute phase difference for 1 811 acentric reflections was 32°. A map produced from these 1 970 reflections, out of 12 346 reflections in the data set, showed a remarkable agreement with the refined structure. Chymosin is many times larger than any molecule whose structure had previously been determined without the use of isomorphous replacement, molecular replacement or anomalous dispersion, and the map demonstrates the potential of maximum-entropy methods in macromolecular structure determination.

### 4.3 Subtilisin BPN*'* and Prosubtilisin

The bacterial serine protease subtilisin BPN’ is widely used as a protein-degrading reagent in household and industrial detergents. The natural enzyme is stabilized in part by the presence of bound calcium at two different sites, a high-affinity site (site A) and a weaker less specific binding site. Site A has been shown to be an impediment to reversible unfolding [38] and complicates its use in detergents containing water-softening agents (metal chelators). A CARB team of scientists headed by Phil Bryan undertook engineering efforts of subtilisin to remove the calcium site A and improve the thermal stability of the resulting modified enzyme. Travis Gallagher and Gary Gilliland carried out the crystallographic studies associated with this project.

**Fig. 3:**
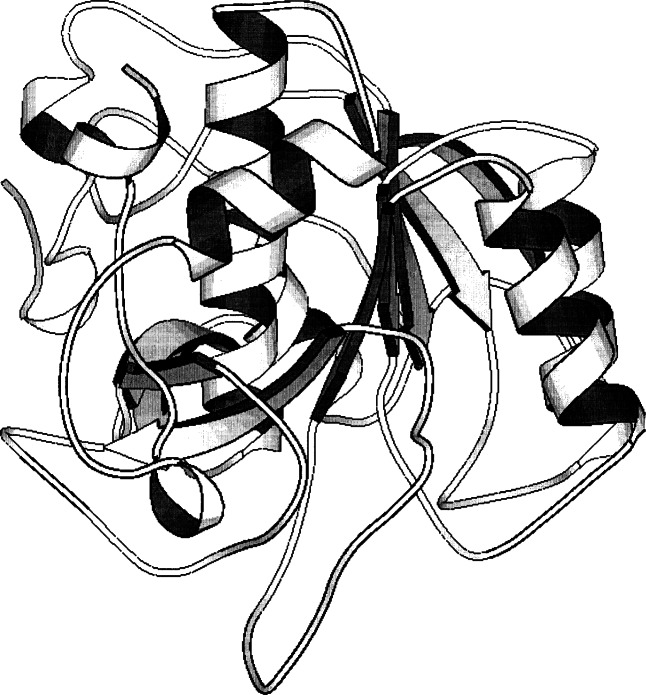
The 1.8 Å structure of engineered subtilisin BPN from *Bacillus amyloliquefaciens* that removed the calcium-binding loop associated with site A. (PDB entry 1sub) [3]. The backbone fold is shown with the α-helix and β-strand secondary structure elements are shown as tightly coiled ribbons and arrows, respectively.

A version of subtilisin BPN*'* lacking site A was produced using genetic engineering methods, and its crystal structure determined at 1.8 Å resolution (see Fig. 3) [3]. This protein structure and the corresponding version containing the site A calcium were compared and analyzed. The helix in the wild type enzyme that is interrupted by the calcium-binding loop is continuous in the deletion mutant. A few residues adjacent to the loop, principally those that were involved in calcium coordination, are repositioned and/or destabilized by the deletion. Because refolding is greatly facilitated by the absence of the Ca-loop, this protein offered a new vehicle for analysis of the folding reaction. Also, at the time this was among the largest internal changes to a protein to be described at atomic resolution.

As suggested above, the mature form of subtilisin is an unusual example of a protein with a high kinetic barrier to folding and unfolding. Removing the calcium-binding site A from subtilisin by deleting amino acids 75–83 greatly accelerated both unfolding and refolding reactions. A disulfide cross-link was introduced between residues 22 and 87 in Δ75–83 subtilisin to probe the conformational entropy of the transition state for folding [39]. The 1.8 Å x-ray structure of this mutant and the effects of the cross-link on the kinetics of unfolding were consistent with an expected loss of entropy of the unfolded protein due to the cross-link, the disulfide accelerates folding relative to the uncross-linked form. The magnitude of the acceleration of folding rate (700 to 850-fold at 25 °C) indicates that residues 22 and 87 are ordered in the transition state such that the disulfide does not affect its total entropy.

The high-resolution crystal structures of four genetically engineered subtilisin BPN*'* variants that vary dramatically in their stability were determined to aid the engineering efforts of the enzyme [40]. The simplest variant contains two altered residues, N218S and S221C. The N218S change was incorporated for its stabilizing effects and its influence on crystallization; the S221C change, a modification of the active site serine, was included to reduce autolysis. The second variant includes two known stabilizing mutations M50F and Y217K. The third variant, in addition to the four single-site mutations, has the Δ75–83 change, removing the high-affinity calcium-binding site. The fourth variant incorporates all of the above changes and two additional site-specific mutations, T22C and S87C that form a stabilizing disulfide bridge.

In summary, extracellular proteases of the subtilisin-class depend upon calcium for stability. Calcium binding stabilizes these proteins in natural extracellular environments, but is an Achilles’ heel in industrial environments that contain high concentrations of metal chelators. Further studies that direct the evolution of calcium-independent stability in subtilisin BPN*'* were carried out [41]. By deleting the calcium binding loop from subtilisin, the enzyme was destabilized, and the analysis of the structure and stability of the loop-deleted prototype followed by directed mutagenesis and selection for increased stability resulted in a subtilisin mutant with native-like proteolytic activity but 1000-times greater stability in strongly chelating conditions.

The folding of the protease subtilisin BPN*'* is dependent on its 77-residue prosegment, which is then auto-catalytically removed to give the mature enzyme. Mature subtilisin represents a class of proteins that lacks an efficient folding pathway. Refolding of mature subtilisin BPN*'* is extremely slow unless catalyzed by the independently expressed prosegment, leading to a bimolecular complex. In order to better understand the role of the prodomain in subtilisin folding, the structure of the processed complex between the prodomain and subtilisin Sbt-70, a mutant engineered for facilitated folding was determined [42–43]. The prodomain is largely unstructured by itself but folds into a compact structure with a four-stranded antiparallel β-sheet and two three-turn α-helices when complexed with subtilisin. The prodomain binds on subtilisin’s two parallel surface α-helices and supplies caps to the N-termini of the two helices. The C-terminal strand of the prodomain binds in the subtilisin substrate-binding cleft. While Sbt-70 is capable of independent folding, the prodomain accelerates the process by a factor of > 10^7^ M^−1^ of prodomain in 30 mM Tris-HCl, pH 7.5, at 25 °C. X-ray structures of the mutant subtilisin folded in vitro, either with or without the prodomain, were compared and showed that the identical folded state is achieved in either case [44]. With knowledge of the prodomain structure five mutations were introduced into the C-terminal region [45]. Analysis of these mutants reveals a general correlation between the ability of the prodomain to bind to native subtilisin and its ability to accelerate subtilisin folding. Later studies were carried out in which the folding equilibrium of the unstable prodomain was shifted by introducing stabilizing mutations generated by design [46]. By sequentially introducing three stabilizing mutations into the prodomain the equilibrium for independent folding was shifted from 97 % unfolded to 65 % folded.

In addition to the protein engineering studies of subtilisin described above a new high-resolution structure of subtilisin BPN*'* was determined. The three-dimensional structure of the serine protease subtilisin BPN*'* has been refined at 1.6 Å resolution in space group C2 to a final *R* value of 0.17. Seventeen regions of discrete disorder were identified and analyzed [47]. Two of these are dual-conformation peptide units; the remainder involves alternate rotamers of side chains either alone or in small clusters. The structure was compared with previously reported high-resolution models of subtilisin BPN*'* in two other space groups. *P*2_1_2_1_2_1_ and *P*2_1_. Apart from the surface, there are no significant variations in structure among the three crystal forms. Structural variations observed at the protein surface occur predominantly in regions of protein-protein contact. The crystal packing arrangements in the three space groups were compared.

## 4.4 Hemoglobin

Collaboration with the Biochemistry Department of the University of Maryland Medical School (Clara Fronticelli and Enrico Bucci) and CARB (Gary Gilliland) was established to characterize natural and variant human hemoglobin through molecular biology, biochemical and crystallographic studies to assess its use as an oxygen carrier for use in blood substitutes. Alterations of the hemoglobin were made to modify two critical properties of hemoglobin, as it exists in solution. The oxygen binding affinity of natural human hemoglobin is too high when it is free in the blood stream (not contained within erythrocytes) because of a lack of allosteric control. The tetrameric protein also dissociates when free in the blood, allowing it to move out of the blood vessels into other tissues reducing its efficacy as an oxygen carrier and creating problems with normal kidney function. The structural studies of the hemoglobin project included the structure determination of natural deoxyhemoglobin and carbonmonoxy hemoglobin [7,51] along with several recombinant variant human hemoglobins [7,52–53]. The structure of T-state sebacyl β_1_Lys82-β_2_Lys82 crosslinked human hemoglobin was also determined [54–56].

The first recombinant human hemoglobin variant, β(V1M+H2Δ), was constructed, characterized and the structure was determined and analyzed [7]. This study also involved collecting x-ray data and refining the structure of natural deoxyhemoglobin using the same protocol as that used for the variant. In this construct the N-terminus was modified to produce one that is similar in its properties to bovine hemoglobin. Analysis of the oxygen binding curves indicates that this mutation results in an additional stabilization of the T-state conformation. In these studies the crystal structure of deoxy-β(V1M+H2Δ) was determined to 2.2 Å resolution and compared with the deoxy structure of natural human hemoglobin. In human deoxyhemoglobin, a sulfate anion is found anchored to the β-chains by a complex network of H-bonds and electrostatic interactions with the N-terminus and βLys82. In the mutant structure, the shortening of the amino-terminal region of the A helix by 1 residue results in the formation of an intrachain electrostatic interaction between the N-terminal amino group and βAsp79. This eliminates the sulfate-binding site, and two water molecules replace the sulfate. At variance with human hemoglobin, the alkaline Bohr effect for β(V1M+H2Δ) is not sensitive to the presence of Cl^−^. This suggests that the sulfate binding site in human hemoglobin also serves as a Cl^−^ binding site, and that the amino-terminal Val1 is essential for oxygen-linked Cl^−^ binding to hemoglobin as well as the Cl^−^-dependent Bohr effect.

The second recombinant hemoglobin variant to be structurally characterized replaced the βVal67 residues with threonines in an attempt to decrease the oxygen affinity. The crystal structure of the mutant deoxyhemoglobin was determined at 2.2 Å resolution [52]. Prior to the crystal structure determination, molecular modeling indicated that the βThr67 side chain hydroxyl group in the distal beta-heme pocket forms a hydrogen bond with the backbone carbonyl of βHis63 and is within hydrogen-bonding distance of the ND atom of βHis63. The mutant crystal structure indicates only small changes in conformation in the vicinity of the βThr67 confirming the molecular modeling predictions. The introduction of threonine into the distal heme pocket, despite having only small perturbations in the local structure, had a marked affect on the interaction with ligands. In the oxy derivative there is a two-fold decrease in O_2_ affinity, and the rate of autoxidation is increased by two orders of magnitude.

In the final study of recombinant hemoglobins, three variants of tetrameric human hemoglobin, with changes at the α_1_β_2_/α_2_β_1_-interface, at the α_1_β_1_/α_2_α_2_-interface, and at both interfaces, were constructed. At α_1_β_2_/α_2_β_1_-interface βCys93 was replaced with alanine, and at the α_1_β_1_/α_2_β_2_-interface the βCys112 was replaced with glycine. The α_1_β_2_ interface variant with βAla93, and the α_1_β_1_/α_2_β_2_ double mutant, containing βAla93 and βGly112, were crystallized in the T-state, and the structures determined at 2.0 Å and 1.8 Å resolution, respectively [53]. A comparison of the structures with that of natural hemoglobin A showed the absence of detectable changes in the tertiary folding of the protein or in the T-state quaternary assembly. At the βGly112 site, the void left by the removal of the cysteine side chain was filled with a water molecule, and the functional characteristics of βGly112 variant were essentially those of human hemoglobin A. At the βAla93 site, water molecules did not replace the cysteine side chain, and the alanine substitution increased the conformational freedom of βHis146, weakening the important interaction of this residue with βAsp94. As a result, when Cl^−^ is present in the solution, at a concentration 100 mM, the Bohr effect of the two mutants containing βAla93 is significantly modified being practically absent below pH 7.4. Based on the crystallographic data, these effects were attributed to the competition between βAsp94 and Cl^−^ in the salt link with βHis146 in T-state hemoglobin. These results point to an interplay between the βHis146-βAsp94 salt bridge and the Cl^−^ in solution regulated by the cysteine present at position β93, indicating yet another role of β93 Cys in the regulation of hemoglobin function.

The crystal structure of human T-state hemoglobin crosslinked with bis(3,5-dibromo-salicyl) sebacate (DecHb) was determined at 1.9 Å resolution [54–55]. The 10-carbon sebacyl residue found in the β-cleft covalently links the two βLys82 residues. The sebacyl residue was found to assume a zigzag conformation with cis amide bonds formed by the NZ atoms of βLys82’s and the sebacyl carbonyl oxygens. When the crosslinked deoxyhemoglobin was compared with deoxyhemoglobin refined using a similar protocol [7], no significant perturbations in the tertiary or quaternary structure were found to be introduced by the presence of the sebacyl residue. However, the conformations of the β_1_Lys82 and β_2_Lys82 are altered because of the crosslinking, and the sebacyl residue displaces seven water molecules in the β-cleft. The carbonyl oxygen that is part of the amide bond formed with the NZ of β_2_Lys82 forms a hydrogen bond with side chain of β_2_Asn139 that is in turn hydrogen-bonded to the side chain of β_2_Arg104. Unexpectedly, the Fe atoms of the α-hemes were found to be oxidized with a water molecule bound [56]. The proximal histidines of the α-subunits move toward the heme plane shifting the F-helix and FG-corner in a manner observed for all other partially oxidized human hemoglobin. This supports the hypothesis that these perturbations may precede the T- to R-state transition. Circular dichroism studies comparing DecHb and natural human hemoglobin in the deoxy and CO ligated forms confirmed that the conformations of the deoxy forms are identical, but the ligated forms have slight differences in the solution structures. DecHb was also found to be more resistant to autoxidation than natural hemoglobin. Thus, the discovery of the oxidation of the alpha-subunits in the deoxy-crystals was quite unexpected. The data confirms that ligation of the α-subunits precedes that of the β-subunits.

As part of the overall hemoglobin effort, the three-dimensional structure and associated solvent of human carboxyhemoglobin was determined at 2.2 Å resolution, and the structure was compared with other R-state and T-state human hemoglobin structures [51]. At the time this structure was solved, it represented the highest resolution human carboxyhemoglobin structure ever determined. The structure is actually a natural variant of hemoglobin. A mutation of the α-subunit, A53S, was discovered during the course of the refinement that forms a new stabilizing crystal contact through a bridging water molecule. The protein structure revealed a significant difference between the α- and β-heme geometries, with Fe-C-O angles of 125° and 162°, respectively. The structure was similar to the earlier reported R-state structures, but there were differences in many side-chain conformations, the presence of a phosphate ion, and the position of the associated water structure. The quaternary changes between the R-state carboxy-hemoglobin and the R2-state and T-state hemoglobin structures were in general consistent with those reported in the earlier structures. The location of a phosphate ion and 238 water molecules in the structure allowed the first comparison of the solvent structures of the R-state and T-state hemoglobin structures. Distinctive hydration patterns for each of the quaternary structures were observed, but a number of conserved water molecule binding sites were found that are independent of the conformational state of the protein.

### 4.5 Glutathione S-Transferase

The glutathione S-transferase studies carried out at CARB have been one of the longest sustained efforts dealing with a single system. The work began as a collaboration between CARB (Gary Gilliland) and Richard Armstrong who, at the time, was a faculty member in the Chemistry and Biochemistry Department of the University of Maryland at College Park. During the course of the experiments, Richard Armstrong spent a sabbatical year at CARB, and he eventually accepted another position at Vanderbilt University. The work was initially focused on one of the isozymes of the mu-class glutathione S-transferase [6, 65–75], but as the work progressed efforts on a number of other glutathione S-transferases from a variety of sources was carried out [76–82]. The work has led to many insights into how the protein structure influences catalysis and its properties.

Glutathione S-transferases are liver detoxification enzymes that catalyze the addition of glutathione to xenobiotic electrophilic compounds, solubilizing them and labeling them for transport to the kidneys for elimination. As mentioned above, a number of crystal structures of glutathione S-transferases in addition to the rat liver, mu-class enzyme have been determined as part of the CARB efforts. These studies resulted in a number of new collaborations with the CARB group. The crystal structure of human alpha-class glutathione S-transferase A1-1 was determined and refined to a resolution of 2.6 Å [67]. This work was done in collaboration with the research group of Alwyn Jones at Uppsala University, Sweden. Next, the three-dimensional crystal structure of glutathione S-transferase of *Schistosoma japonicum* fused with a conserved neutralizing epitope on gp41 of human immunodeficiency virus type 1 (HIV-1) was determined at 2.5 Å resolution [73–74]. These studies were carried out in collaboration with Dan Carter’s research group that was then at Marshall Space Flight Center, Alabama. The three-dimensional structure of the sigma-class glutathione S-transferase in complex with the product 1-(S-glutathionyl)-2,4-dinitrobenzene was solved by multiple isomorphous replacement techniques to a resolution of 2.4 Å [75–76,78]. This work was carried out in collaboration with the Armstrong group and a group at NIH headed by J. Piatigorsky. Most recently, complexed structures of a naturally occurring variant of human pi-class glutathione S-transferase isozyme 1-1 with either S-hexylglutathione or (9R,10R)-9-(S-glutathionyl)-10-hydroxy-9, 10-dihydrophenanthrene bound at the active site were determined at resolutions of 1.8 Å and 1.9 Å, respectively [79]. These structures were done in collaboration with Xinhua Ji who moved after completing his postdoctoral studies at CARB to a new position as head of his own structural biology group at the National Cancer Institute in Frederick. Below, a number of the highlights of the structural investigation of the rat liver mu-class glutathione S-transferase are presented.

**Fig. 4:**
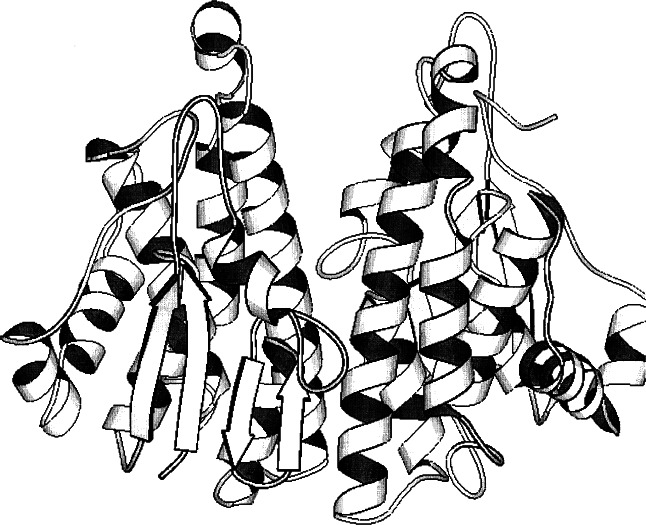
The 2.2 Å structure of rat liver glutathione S-transferase with bound glutathione (not shown) (PDB entry 6gst) [6,77]. The backbone fold is shown with the α-helix and β-strand secondary structure elements are shown as tightly coiled ribbons and arrows, respectively.

The crystal structure of a mu class glutathione S-transferase from rat liver in complex with the physiological substrate glutathione (GSH) was solved to 2.2 Å resolution by the method of multiple isomorphous replacement (see Fig. 4) [6]. Site-specific mutagenesis played an important role in the solution of the structure in that the cysteine mutants C86S, C114S, and C173S were used to help locate the positions of heavy atoms and to align the sequence with the model derived from the experimental phases. The final model consisted of the complete polypeptide chains of the monomers composed of 434 amino acid residues, two GSH molecules, and 474 water molecules. The structure of the enzyme subunit can be divided into two domains separated by a short linker, a smaller α/β domain (domain I, residues 1–82), and a larger α domain (domain II, residues 90–217). Domain I contains four β-strands which form a central mixed β-sheet and three α-helices which are arranged in a βαβαββα motif that functions as the glutathione domain. Domain II is composed of five α-helices and appears to be primarily responsible for xenobiotic substrate binding.

Unexpectedly, it was discovered from the structure that Tyr6 stabilized the thiolate intermediate of the glutathione during catalysis [6,65–66]. The role of the hydroxyl group of Tyr6 in the catalytic mechanism of isoenzyme 3-3 of rat glutathione S-transferase was examined by x-ray crystallography and site-specific replacement of the residue with phenylalanine and evaluation of the catalytic properties of the mutant enzyme. The structure of the binary complex of the enzyme and glutathione indicates that the hydroxyl group of Tyr6 is located between 3.2 Å and 3.5 Å from the sulfur of glutathione, well within hydrogen bonding distance. Removal of the hydroxyl group of Tyr6 has no effect on the dissociation constant for glutathione. Nevertheless the Y6F mutant exhibits a turnover number which is only about 1 % that of the native enzyme. The structural results and experimental characterization of the Y6F mutant suggest that the hydrogen bond between Tyr6 and the enzyme-bound nucleophile helps to lower the pK_a_ of GSH in the binary enzyme-substrate complex.

During the structural analysis it was noticed that Thr13 forms an on-face hydrogen bond with Tyr6. This led to the postulate that it might influence catalysis through what are known as second-sphere interactions [69]. A number of site-directed variants were constructed, characterized, crystallized, and structures determined [77]. Removal of the second-sphere influence of the on-face hydrogen bond between the hydroxyl groups T13 as in the T13V and T13A mutants elevates the pK_a_ of enzyme-bound GSH by about 0.7 pK_a_ units. Crystal structures of these variants show minor structural changes in the active site and suggest the changes in pK_a_ of are due to the presence or absence of the on-face hydrogen bond. The T13S mutant has a completely different side-chain hydrogen-bonding geometry than T13 in the native enzyme and catalytic properties similar to the T13A and T13V mutants consistent with the absence of an on-face hydrogen bond. The side chain methyl group of T13 is essential in enforcing the on-face hydrogen bond geometry and preventing the hydroxyl group from forming other more favorable conventional hydrogen bonds.

Further investigation of the enzymatic mechanism of the mu-class glutathione S-transferase led to the structure determation of enzyme complexes with a transition-state analogue, 1-(S-glutathionyl)-2,4,6-trinitrocyclohexadienate, and a product, 1-(S-glutathionyl)-2,4-dinitrobenzene, of a nucleophilic aromatic substitution (SNAr) reaction at 1.9 Å and 2.0 Å resolution, respectively [70]. The active sites of the two structures, which were quite different, represented snapshots along the reaction coordinate for the enzyme-catalyzed reaction of glutathione with 1-chloro-2,4-dinitrobenzene and revealed specific interactions between the enzyme, intermediate, and product that are important for catalysis. The geometries of the intermediate and product were used to postulate reaction coordinate motion during catalysis.

The structures of two other product complexes led to a quite a startling discovery, the enzyme amino acid residues that participated in catalysis could vary depending upon the structure of the substrate [72]. The analysis of the crystal structures of the rat liver mu-class glutathione S-transferase complexed with the products (9R,10R)- and (9S,10S)-9-(S-glutathionyl)-10-hydroxy-9,10-dihydrophenanthrene at resolutions of 1.9 Å and 1.8 Å, respectively, provided new clues to the enzymes catalytic behavior. The hydroxyl group of Tyr115 was found hydrogen-bonded to the 10-hydroxyl group of (9S,10S)-2, a fact suggesting that this residue could act as an electrophile to stabilize the transition state for the addition of GSH to epoxides. As it turns out, the Tyr115Phe mutant isoenzyme 3-3 is about 100-fold less efficient than the native enzyme in catalyzing the addition of GSH to phenanthrene 9,10-oxide and about 50-fold less efficient in the Michael addition of GSH to 4-phenyl-3-buten-2-one. The side chain of Tyr115 is positioned so as to act as a general-acid catalytic group for two types of reactions that would benefit from electrophilic assistance.

In further investigations of the mechanism of the mu-class glutathione S-transferase, the tyrosines in the enzyme were globally substitute 3-fluorotyrosine and the structure determined at 2.2 Å resolution [80,82]. The structure of the tetradeca-(3-fluorotyrosyl) M1-1 glutathione S-transferase (3-FTyr GST) was the first x-ray crystal structure with complete substitution of tyrosine with 3-fluorotyrosine. Although fluorinated amino acid residues have frequently been used in biochemical and NMR investigations of proteins, no structure of a protein that has been globally substituted with a fluorinated amino acid had previously been reported. Numerous conformational changes were observed in the protein structure as a result of substitution of 3-fluorotyrosine for tyrosine. The results of the comparison of the crystal structure of the fluorinated protein with the native enzyme revealed conformational changes for most of the 3-fluorotyrosines. The largest differences were seen for residues where the fluorine, the OH, or both are directly involved in interactions with other regions of the protein or with a symmetry-related molecule. The fluorine atoms of the 3-fluorotyrosine interact primarily through hydrogen bonds with water molecules and other residues. In several cases, the conformation of a 3-fluorotyrosine is different in one of the monomers from that observed in the other, including different hydrogen-bonding patterns. Altered conformations of the residues can be related to differences in the crystal packing interactions of the two monomers in the asymmetric unit. The fluorine atom on the active-site Tyr6 is located near the S atom of the thioether product (9R,10R)-9-(S-glutathionyl)-10-hydroxy-9,10-dihydrophenanthrene and creates a different pattern of interactions between 3-fluorotyrosine 6 and the S atom. Studies of these interactions helped explain why 3-FTyr GST exhibits spectral and kinetic properties distinct from the native GSH transferase.

A second structural study of glutatione S-transferase was undertaken with 5-fluorotryptophan substituted for tryptophan [81]. This structure represents the first of a protein substituted with 5-fluorotryptophan, two substitutions in each subunit. The crystal structure of the 5-fluorotryptophan-containing enzyme was solved at a resolution of 2.0 Å by difference Fourier techniques. The structure reveals local conformational changes in the structural elements that define the approach to the active site. The changes are attributed to steric interactions of the fluorine atoms associated with 5-FTrp146 and 5-FTrp214 in domain II. These changes appear to result in the enhanced rate of product release.

## 5. Current NIST Macromolecular Crystallography Studies

Currently macromolecular crystallographic studies are focused in two major areas, the enzymes in the chorismate pathway [9,93–94] and structural genomics [10,95–96]. Jane Ladner at CARB and Travis Gallagher at the NIST main campus are carrying out the chorismate enzyme studies. The structural-genomics effort is a joint project between the NIST (headed by Gary Gilliland) and University of Maryland (headed by John Moult, John Orban and Osnat Herzberg) principal investigators at CARB, and the group of Andrew Howard at the Illinois Institute of Technology and the Advanced Photon Source at Argonne National Laboratory. One spinoff from the structural genomics work, the discovery of cryosalts [97], will be described below.

### 5.1 Chorismate Pathway Enzymes—Metablic Engineering

**Fig. 5:**
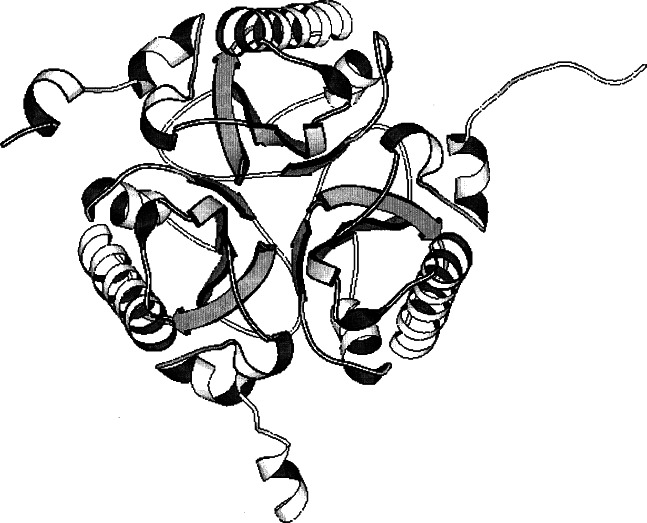
The 1.3 Å structure of the *Bacillus subtilis* chorismate mutase catalytic homotrimer (PDB entry 1dbf) [9]. The backbone fold is shown with the α-helix and β-strand secondary structure elements are shown as tightly coiled ribbons and arrows, respectively.

The structural investigation of the shikimate or chorismate enzymes are a part of a large-scale Biotechnology Division project to provide a generic description of carbon flow and energy utilization in chorismate metabolic pathways by measuring reaction properties, modeling the mechanisms of chemical transformations, characterizing enzyme structures, and mapping pathway control nodes involved in the biocatalytic conversion of glucose to aromatic hydrocarbons. These pathways are of intense interest since they offer routes to the biosynthesis of high-value biotechnology products. The first structural efforts focused on chorismate mutase from *Bacillus subtilis* [9]. Chorismate mutase catalyzes the rearrangement of chorismate to prephenate that can subsequently be converted to aromatic products such as tyrosine or phenylalanine. A new orthorhombic crystal form of the enzyme was found during the crystallization trials and x-ray data was collected to 1.3 Å resolution. The final coordinates of the structure that was solved by molecular replacement are composed of three complete polypeptide chains of 127 amino acid residues. In addition, there are 9 sulfate ions, 5 glycerol molecules and 424 water molecules clearly visible in the structure (see Fig. 5). A glycerol molecule and sulfate ion in each of the active sites was found mimicking a transition state analog. In this structure, the C-terminal tails of the subunits of the trimer are hydrogen bonded to residues of the active site of neighboring trimers in the crystal, and thus, cross-link the molecules in the crystal lattice. This cross-linking may help to account for the much-improved quality of diffraction of this crystal form. The results of this work have supported ongoing computational chemistry studies investigating the mechanism. The mechanism of the enzyme-catalyzed rearrangement is not known.

The second enzyme of this pathway for which the structure has been solved to high resolution is chorismate lyase from *Escherichia coli*. The enzyme choris-mate lyase catalyzes the removal of pyruvate from chorismate to produce 4-hydroxy benzoate for the ubiquinone pathway. The enzyme has been crystallized in four distinct forms, three of which have been characterized by x-ray diffraction [93]. Surprisingly, all four crystal forms grow from the same chemical conditions. The wild-type enzyme tends to aggregate, even in the presence of reducing agent, and yielded only one crystal form (monoclinic, form 1) that grew in intricate clusters. Chemical modification of the cysteines mitigated problems with aggregation and solubility, but it did not affect crystal growth behavior. Converting the enzyme’s two cysteines to serines largely eliminated protein aggregation. The double mutant retains full enzymatic activity and crystallizes in three new forms, one of which (triclinic) diffracts to 1.1 Å resolution. Chorismate lyase is monomeric in *Escherichia coli* consisting of 164 residues. The structures of the wild-type enzyme the active double Cys-to-Ser variant complexed with product were determined at 2.0 Å and 1.4 Å, respectively [94]. The protein fold involves a six-stranded an-tiparallel β-sheet with novel connectivity. The product is bound internally, adjacent to the sheet, with its polar groups coordinated by two main-chain amides and by the buried side-chains of Arg 76 and Glu 155. The 4-hydroxy benzoate is completely sequestered from solvent in a largely hydrophobic environment behind two helix-turn-helix loops. The extensive product binding that is observed is consistent with biochemical measurements of slow product release and 10-fold stronger binding of product than substrate. Substrate binding and kinetically rate-limiting product release apparently require the rearrangement of these active-site-covering loops.

### 5.2 Structural Genomics

A large portion of the gene products of completely sequenced organisms are of completely unknown function or hypothetical and cannot be related to any previously characterized proteins. Structural studies provide one means of obtaining functional information in these cases. CARB scientists have undertaken a structural genomics project aimed at determining the structures of 50 hypothetical proteins from *Haemophilus influenzae* to aid in the elucidation of their function [10]. In the development of an effective structural genomics program, target selection, protein production, crystallization, structure determination, and structure analysis must all make use of recent advances in technology to streamline procedures. Early results from this and similar projects are encouraging in that some level of functional understanding can be deduced from experimentally solved structures. Below the results of two of a number of structures that have been solved are described.

In the first case, a hypothetical protein encoded by the gene YjeE of *Haemophilus influenzae* was selected as one of the targets for the structural genomics project, for x-ray analysis to assist with the functional assignment [95]. The protein is considered essential to bacteria since the gene is present in virtually all bacterial genomes, but not in those of archaea or eukaryotes. The amino acid sequence shows no homology to other proteins. However, the presence of the Walker A motif G-X-X-X-X-G-K-T indicates the possibility of a nucleotide-binding protein. The YjeE protein was cloned, expressed, and the crystal structure determined by the Mutiwavelength Anomalous Dispersion method at 1.7 Å resolution. The protein has a nucleotide-binding fold with a P-loop typical of many ATPases and GTPases, although the topology of the β-sheet is unique. Crystallization experiments and nucleotide modeling indicate the preference of YjeE for ATP rather than for GTP. The observation of a hydrolyzed nucleotide (ADP) in the active site implies ATPase activity of YjeE. Structural comparison of YjeE with the P-loop proteins from the 14 known families shows that it represents a new class of P-loop ATPases. The phylogenetic pattern of YjeE strongly suggests its involvement in cell wall biosynthesis. The protein is likely to be an ATP-dependent regulator of peptidoglycan metabolism given the distribution of conserved residues and structural features typical for “molecular switches”. As such, it may be a promising target for new antibiotics.

The second case is similar to the first, in that a hypothetical protein encoded by the gene YacE gene of *Haemophilus influenzae* was selected as one of the targets for the structural genomics project, for x-ray analysis to assist with the functional assignment [96]. However, during the structural analysis, functional assignment of YacE as a dephospho-coenzyme A kinase was reported [98]. The assignment was based on the enzyme assay and reaction product characterization of the homologous protein from *Escherichia coli*. Dephospho-coenzyme A kinase catalyzes the final step in CoA biosynthesis, the phosphorylation of the 3*'*-hydroxyl group of ribose using ATP as a phosphate donor. The structure of the protein from *Haemophilus influenzae* was determined at 2.0 Å resolution in complex with ATP [96]. The protein molecule consists of three domains: the canonical nucleotide binding domain with a five-stranded parallel β-sheet, the substrate-binding α-helical domain, and the lid domain formed by a pair of α-helices. The overall topology of the protein resembles the structures of nucleotide kinases. ATP binds in the P-loop in a manner observed in other kinases. The CoA binding site is located at the interface of all three domains. The double-pocket structure of the substrate-binding site is unusual for nucleotide kinases. Amino acid residues involved in substrate binding and catalysis have been identified. The structure analysis suggests large domain movements during the catalytic cycle.

#### 5.2.2 Cryosalts

Quality data collection for macromolecular cryocrystallography requires suppressing the formation of crystalline or microcrystalline ice that may result from flash-freezing crystals. During the course of the structural genomics studies at CARB, a number of problems arose in which flash-freezing using traditional cryosolvents such as glycerol was ineffective [97]. A number of non-traditional approaches for solving this problem were tried. It was discovered that the use of lithium formate, lithium chloride, and other highly soluble salts were effective in forming ice-ring-free aqueous glasses upon cooling from ambient temperature to 100 K. The aqueous glass-forming properties of highly soluble salts have been known for many years. Nevertheless, these compounds had not been reported as cryoprotectants for macromolecular crystallography. Highly soluble salt or cryosalt addition to commonly used crystallization solutions and protein crystals induce glass formation under typical conditions for cryocrystallography with attributes comparable to the traditional organic cryoprotectants. In addition, the absence of deleterious effects on mosaicity and diffraction resolution of cryosalt-treated crystals makes them as useful as the more traditional cryoprotectants.

## 6. Structural Biology Databases

NBS/NIST has been very active in the development and distribution of structural biology databases since Biomolecular Structure Group of the Chemical Thermodynamics Division of the Center for Chemical Physics at NBS was created. Initially efforts were focused on developing a novel resource, the Biological Macro-molecule Crystallization Database to assist in the production of crystals required for x-ray crystallographic studies [11,99–105]. More recently the NIST Biotechnology Division has been involved in establishing the Research Collaboratory for Structural Bioinformatics that has been successful at acquiring the Protein Data Bank that is jointly funded by NSF, NIH, and DOE [12,106–109].

### 6.1 Biological Macromolecule Crystallization Database

The NIST/CARB Biological Macromolecule Crystallization Database (BMCD) contains the crystallization and crystal data on all forms of biological macromolecules that have produced crystals suitable for x-ray diffraction studies [11]. Despite the more than fifty years of experience in the production of diffraction quality crystals, there are no predictive methods for determining the crystallization behavior of biological macromolecules. Thus, the motivation for the creation of the BMCD was to provide comprehensive information to facilitate the development of crystallization strategies to produce large single crystals suitable for x-ray structural investigations [100].

The BMCD has its beginnings in the late 1970s and early 1980s in work that was initiated in Dr. David Davies’s laboratory at NIH [110]. In 1987, with assistance from the NIST Standard Reference Data Program, the data were incorporated into a true database and distributed with software that made it accessible using a personal computer [11,99]. The database was released to the public in 1989 as the NIST/CARB (Center for Advanced Research in Biotechnology) Biological Macromolecule Crystallization Database, Version 1.0. In 1990 a second version of the software and data for the PC database was released [100], and in 1994 the BMCD began including data from crystal growth studies carried out in microgravity [101–104]. Recently, the BMCD has been ported to a UNIX platform and made web-based to take advantage of the development of network capabilities that gives the user community access to the most recent updates and allows rapid implementation of new features and capabilities of the software [105].

### 6.2 Protein Data Bank

The Protein Data Bank (PDB) is the single international archive of biological macromolecular structures [12]. The Rutgers, NIST, and UCSD San Diego Super Computer Center members of the Research Collaboratory for Structural Bioinformatics (RCSB; http://www.rcsb.org/) has been fully responsible for its management since July 1, 1999 [12,106–109]. The archive is growing at a rapid rate; in addition, the complexity of structures continues to increase. Several ribosomal subunits have been deposited and released in 2001. The structure of the large subunit of the ribosome, which includes 2833 RNA nucleotides and 27 proteins, was released in August. At the end of 2001, there have been nearly 17,000 structures deposited in the PDB. The demographics of the current holdings are shown at http://www.rcsb.org/pdb/holdings.html.

The access and distribution of the archival data is through the primary Website at UCSD and through mirrors located at Rutgers University, NIST, and in other locations throughout the world. The PDB receives an average of 115 000 hits per day on the primary Web site alone. The PDB Web sites provide users with direct query and reporting capabilities using the underlying databases. Query across the complete PDB has nevertheless been limited by missing, erroneous, and inconsistently reported experimental data, nomenclature, and functional annotation. This inconsistency reflects the evolution of experimental methods, functional knowledge of proteins, and methods used to process these data over the years. NIST has been involved in improving data uniformity since the RCSB assumed its PDB management responsibilities [107]. It has done so in two ways. The first is file-by-file processing in which files of a particular family of proteins are processed individually using many of the software tools that the annotators use in processing new entries. The second approach is curating data values for a particular data item from all files. The efforts at NIST in collaborative efforts with the other centers have substantially increased the reliability of queries of the PDB database. The data uniformity efforts have recently been used to generate a complete set of PDB entries in the mmCIF format. These are currently available as a beta test files via ftp at ftp://beta.rcsb.org/pub/pdb/uniformity/data/mmCIF/ [109].

## Footnotes

1 Certain commercial equipment, instruments, or materials are identified in this paper to foster understanding. Such identification does not imply recommendation or endorsement by the National Institute of Standards and Technology, nor does it imply that the materials or equipment identified are necessarily the best available for the purpose.
